# Transforming Cl-Containing Waste Plastics into Carbon Resource for Steelmaking: Theoretical Insight

**DOI:** 10.1021/acsengineeringau.3c00021

**Published:** 2023-09-15

**Authors:** M. Hussein N. Assadi, Esmail Doustkhah

**Affiliations:** † RIKEN Center for Emergent Matter Science (CEMS), 2-1 Hirosawa, Wako, Saitama 351-0198, Japan; ‡ School of Materials Science and Engineering, The University of New South Wales, Sydney, New South Wales 2052, Australia; ¶ Koç University Tüpraş Energy Center (KUTEM), Department of Chemistry, Koç University, Istanbul 34450, Turkey

**Keywords:** ab initio, molecular dynamics, steelmaking, recycling, waste plastics, sustainability, polymer upconverting

## Abstract

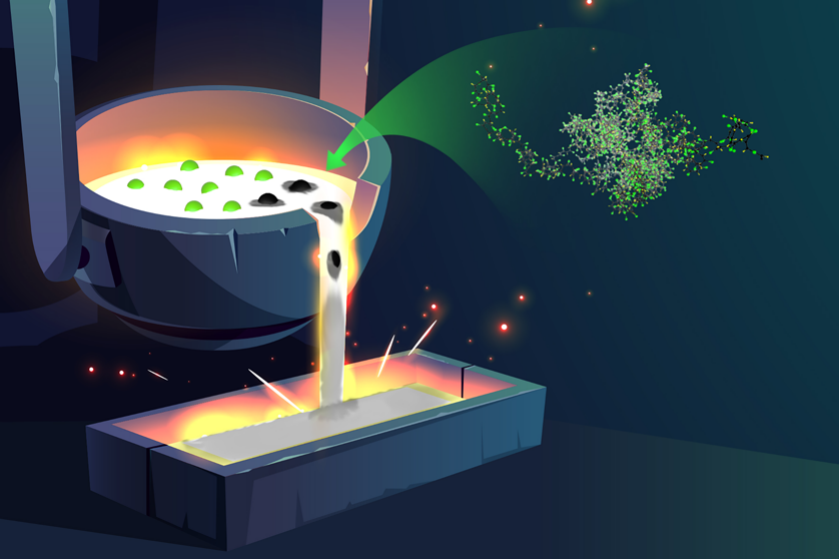

The accumulation of waste plastics poses a significant environmental challenge, leading to persistent pollution in terrestrial and aquatic ecosystems. A practical approach to address this issue involves the transformation of postconsumer waste plastics into industrially valuable products. This study focuses on an example of harnessing the carbon content in these polymers for carbon-demanding industrial processes, thereby reducing waste plastics from the environment and alleviating the demand for mined carbon resources. Employing quantum simulations, we examine the viability of polychloroprene as a carburizing agent in the steelmaking process. Our simulations reveal that polychloroprene exhibits excellent carbon diffusivity in molten iron, with a theoretical diffusion coefficient of 8.983 × 10^–5^cm^2^ s^–1^. This value competes favorably with that of metallurgical coke and surpasses the carbon diffusivity of other polymers, such as polycarbonate, polyurethane, and polysulfide. Additionally, our findings demonstrate that the chlorine content in polychloroprene does not permeate into molten iron but instead remains confined to the molten iron and slag interface.

## Introduction

Polymers are ubiquitous in modern life because of their ease of fabrication, moldability, low cost, and resistance to harsh conditions.^1−3^ However, since most polymers are not biodegradable, their waste aggregates in the ecosystem, resulting in one of the most severe environmental challenges ever. In 2021, the annual plastic production, with a 4% year-on-year rise, reached 390.7 million tons.^4^ Out of this, according to the *Minderoo Foundation*, a total of 139 million tons of waste was generated from single-use plastic products.^5^ This figure signifies an increase of 6 million tons compared to the amount produced in 2019. Although, in principle, most plastic waste can be industrially recycled, in practice, the rules and policies regarding the collection, sorting, and recycling of plastic waste are often loose. As a result, much of the waste plastics is eventually released into the oceans, rivers, and landfills. Recent studies show that less than 10% of waste plastics are recycled.^6^ Therefore, policies and measures as well as research and development should be improved to promote a greater recycling rate.

**Figure 1:**
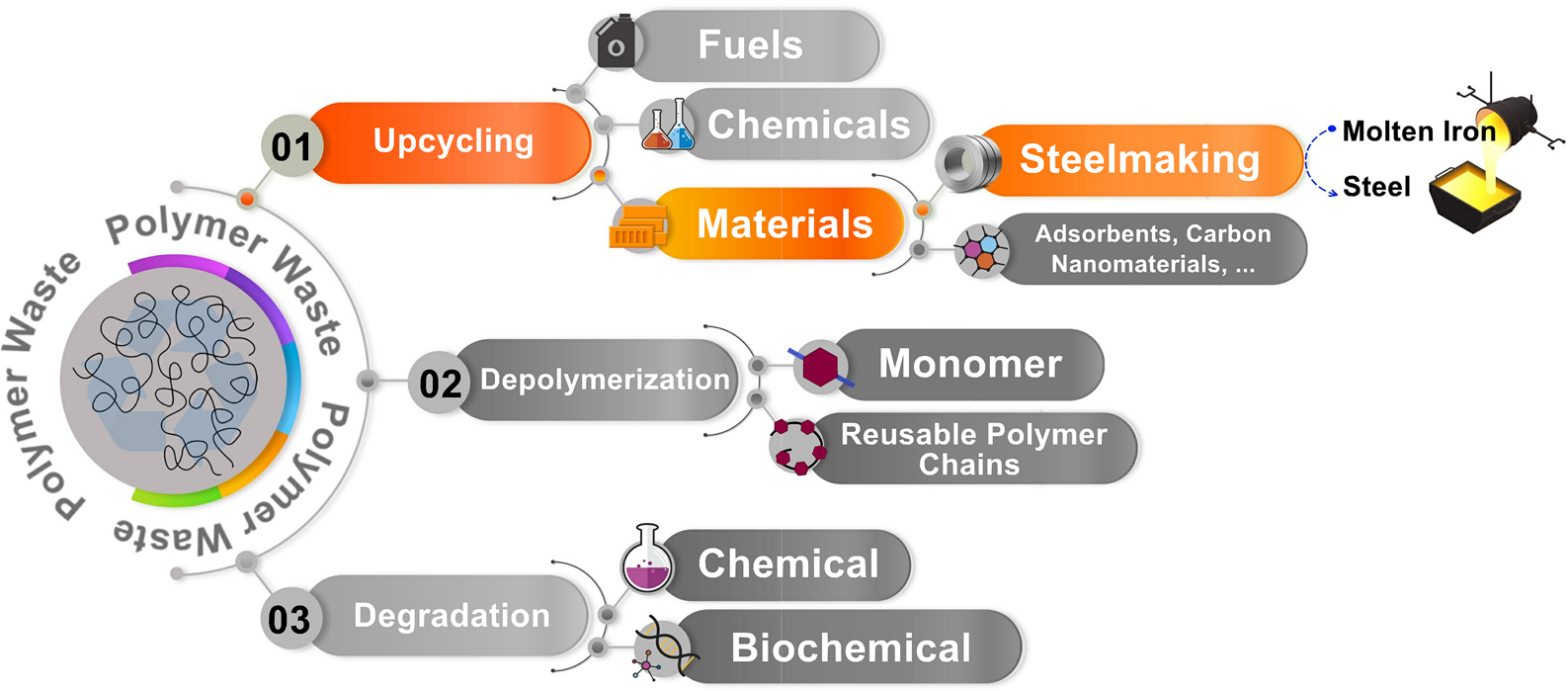
Schematic representation of the major possible methods for utilizing waste plastics in various industries. These strategies reduce the volume of waste plastics in landfills and the oceans.

For polymers that cannot be recycled using mechanical or thermal methods, there are three main options: depolymerization, degradation, and upcycling, all schematically shown in Figure 1. Depolymerization revives the precious monomers or polymer chains that could later be utilized in the same polymer fabrication industry.^7^ In this case, the polymer undergoes relative depolymerization into polymer chains (e.g., removing cross-linked bonds in rubber through devulcanization^8^) or a complete conversion into corresponding monomers by using a catalyst or treatment under specific conditions.^9,10^ This strategy is promising, but the depolymerization process can sometimes be costly and not economical for low-cost polymers.

Polymer degradation represents a highly effective approach for removing plastic waste, which can be categorized into two primary classes: chemo-degradation and biodegradation methods. Unlike upcycling method, this method cannot form valuable materials showing promising adsorption or catalytic activities.^11,12^ Chemical methods predominantly involve oxidation and hydrolysis, which may necessitate significant energy and oxidizing agent consumption. Nevertheless, these methods ultimately result in the mineralization of the polymer and the production of less toxic or nontoxic compounds. Like other degradation processes for waste materials, while the output of degradation is environmentally benign, it does not lead to efficient product formation in terms of element economy and energy utilization.^13−15^ Moreover, the degraded products may not always be environmentally friendly on a large scale and in the long term. For instance, microbial degradation can produce methane, which may have adverse ecological implications.^16,17^

More particularly, the upcycling of less precious polymers can be an excellent solution to valorize the waste into wealth with a value-added strategy.^18^ This method provides valuable carbon-based products such as fuels and carbon structures (e.g., carbon nanotube), which can be utilized in several industries.^19^ The use of such pyrolyzed polymers in the metallurgy industry for steelmaking by mixing carbonized products with molten iron is one example that is widely investigated. The main concern is using a polymer whose heteroatoms are not detrimental to the standard steelmaking process.

Accordingly, in recent years, waste plastics have attracted attention as a supplementary carbon source in the steelmaking industry, partially substituting for metallurgical coke and natural coal. Such use is especially attractive in electric arc furnaces where carbonaceous solids are directly injected into slag to reduce the iron oxide in rusty scraps and to carburize the molten iron.^20^ Accordingly, substituting natural carbon sources for waste plastics is relatively straightforward. However, to ensure quality, the iron/slag interfacial interactions must be well understood and carefully controlled.^21^

One issue that needs careful consideration is the effect of elements other than carbon within waste plastics. These elements may diffuse in molten iron or remain in the slag. Some plastics’ interactions with molten iron have been experimentally characterized—such as rubber, high-density polyethylene, and Bakelite.^22^ However, the suitability of chlorine-bearing polymers remains an open question. The element Cl is corrosive to the plant and produced steel; therefore, understanding its dynamics during the polymer decomposition process is paramount. Cl in steel and steelmaking processes is a less commonly studied element. So far, it is generally accepted that Cl impurities, at a few parts per billion,^23^ can occur in steel grains and more so in steel’s grain boundaries.^24^ Higher Cl concentrations, however, are detrimental to the metal’s ductility^25^ and require dedicated treatment for chlorine removal.^26^

In this work, we use quantum chemistry simulations to examine the interfacial reactions between molten iron and polychloroprene as a representative of Cl-bearing plastics. We investigate the carburizing potential of polychloroprene in steelmaking and monitor Cl’s whereabouts during the decomposition of the polymer. Polychloroprene, also known as neoprene, is a prevalent and low-cost polymer mainly used in electric insulators, adhesives, and construction materials. Therefore, any new recycling and recovery strategies would reduce the amount of this polymer in the landfill.^27^ The insight provided here would facilitate the use of waste plastics in the steelmaking industry by demonstrating polychloroprene’s suitability.

## Settings and Models

**Figure 2:**
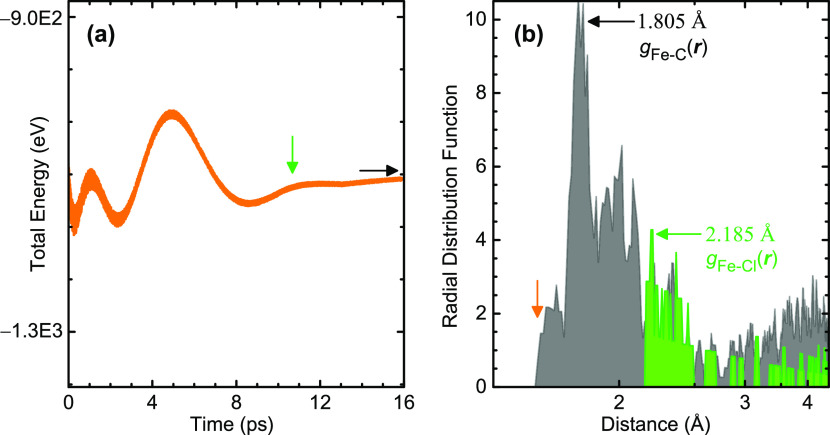
(a) Energy fluctuations during the *ab initio* molecular dynamics run throughout the simulation. The energy smoothly converged to a constant value (marked with a black arrow) after *t* = ∼10.003 ps (marked with a green arrow). (b) Calculated partial radial distribution function *g*(***r***) for Fe– C and Fe–Cl pairs. The *g*(***r***) graph was calculated at 200 points per Å.

The decomposition, dissolution and diffusion processes were simulated using *ab initio* molecular dynamics, as implemented in the VASP package,^28,29^ within the canonical ensemble (NVT) using the Nosé–Hoover thermostat.^30,31^ The temperature was kept constant at 1823.15 K (1550 °C), iron’s melting point at ambient pressure. The simulation was carried out for 16 ps with time steps of 0.5 fs. As marked with a green arrow in Figure 2a, in the last ∼6 ps of the simulation, the energy fluctuations dropped below 0.1% of the total value. The total energy also started approaching a constant value, marked with a black arrow. These conditions indicate equilibrium. Electronically, the density functional calculations were performed using projector-augmented wave (PAW) potentials^32^ and generalized gradient approximation (GGA),^33^ while only the Γ point was used for Brillouin zone sampling. The GGA functional is specifically accurate in describing metallic systems.^34^

The energy convergence threshold for each density functional was set at 10^–5^ eV. The pseudopotentials contained the following electrons: 3*p*^6^3*d*^7^4*s*^1^ for Fe, 2*s*^2^2*p*^2^ for C, and 3*s*^2^3*p*^5^ for Cl. The dissolved monomers were constructed so they contained no hydrogen, as at the simulation temperature, H is too volatile to stay at the polymer/molten iron interface.^35,36^ These simulation settings were previously shown to adequately produce molten iron’s pair correlation function and predict polymers’ atomistic behavior at high temperatures.^35,37^ Moreover, NVT molecular dynamics have proven to be a robust predictive tool in studying solid/liquid interfaces of molten iron.^38,39^

## Results and Discussion

**Figure 3:**
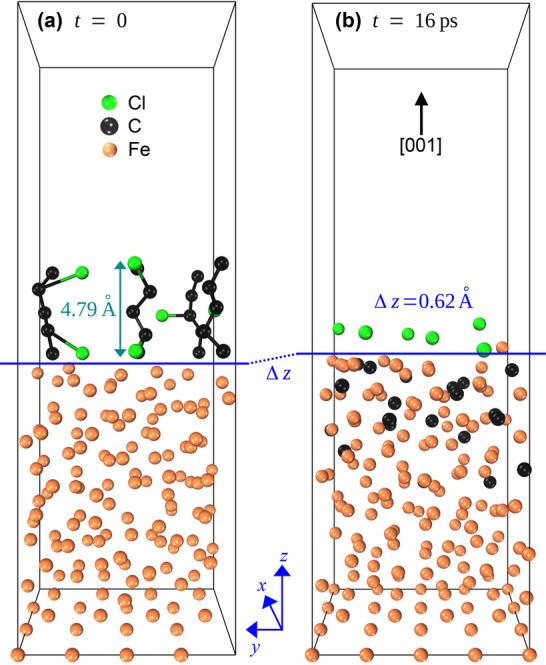
(a) Initial configuration of polychloroprene on the molten iron surface at the start of the molecular dynamics run at *t* = 0. (b) Decomposition and dissolution of polychloroprene at *t* = 16 ps. Only carbon was found to be diffused in molten iron. The final structure was obtained by averaging the coordinates of each atom in the last 100 frames of the molecular dynamics run. The averaging process was necessary as any single snapshot of the molecular dynamics run might have contained an atom at random and odd coordinates not representative of the usual position of that atom. Such unrepresentative coordinates could be caused by the random motion of atoms at the high temperature of the simulation or by numerical fluctuations.

For simulating the molten iron surface, a 3*a* × 4*a* × 5*a* supercell of αFe’s conventional unit cell (*a* = 2.867 Å) was constructed and cleaved along the long axis, creating an interface of 98.595 Å^2^ area. The Fe slab was 11 atomic layers deep and contained 132 Fe ions in total. The atoms at the bottom Fe layer (*z* = 0) were fixed to their coordinates to confine the diffusion process to one side of the interface, which is the case in reality. After adding an ample vacuum slab of 20 Å, this structure was then equilibrated at *T* = 1823.15 K, resulting in a liquidlike Fe state. The pair correlation function of this structure was previously shown to resemble that of molten iron.^35^ We then brought two chloroprene monomers, containing 20 and 6 Cl atoms, to the molten iron’s surface. Under the applied periodic boundary conditions, these monomers mimic the structure of the polychloroprene polymer. The disposition of these monomers on molten iron was carried so that no two atoms at the interface were closer than ∼1.5 Å. Otherwise, the convergence of electronic minimization would have been impossible. This structure, shown in Figure 3a, constitutes the beginning of the dissolution process.

Equilibrating the initial structure of Figure 3a for 16 ps results in the final structure in Figure 3b. Here, the chloroprene monomers are completely decomposed as none of the initial bonds that held the monomers are intact any more. Furthermore, we can see that the C atoms have left the molten iron’s surface and diffused into the Fe’s inner structure. However, in contrast to C, Cl atoms remained above the molten iron interface, showing no sign of diffusion. To further investigate the strikingly different C and Cl behaviors at the molten iron surface, we analyze the partial radial distribution function *g*(***r***) of Fe–C and Fe–Cl pairs, shown in Figure 2b. Partial *g*(***r***) quantifies the likelihood of finding C and Cl at different distances from a given Fe atom.

According to Figure 2b, *g*_Fe–C_(***r***) shows its first nonzero values at 1.645 Å (orange arrow) and peaks at 1.805 Å. Furthermore, *g*_Fe–C_(***r***) shows continuous nondiminishing values at larger distances and a secondary broad peak around ∼4 Å. This *g*(***r***) behavior demonstrates the thorough mixing of Fe and C, where most C atoms are at a distance of ∼1.8 Å from the closest Fe atoms. At the same time, some C atoms breached closer due to the rotational and translational motion of the molten iron atoms. The most probable distance between Fe and C, ∼1.8 Å, is very close to the Fe – C bond length in cementite, Fe_3_C, which is 1.9 Å,^40^ indicating a high probability of Fe – C bond formation if the molten iron were cooled, forming carbon steel. In contrast, *g*_Fe–Cl_(***r***) was zero up to *r* = ∼ 2.185 Å, where it started with its maximum value. *g*_Fe–Cl_(***r***), taking discrete values at higher distances, declined rapidly with distance, indicating that Cl atoms were confined to a narrow space at the interface. In short, *g*(***r***) analysis shows the exclusive diffusion of C atoms into the molten iron and the accumulation of Cl atoms at the interface.

**Figure 4:**
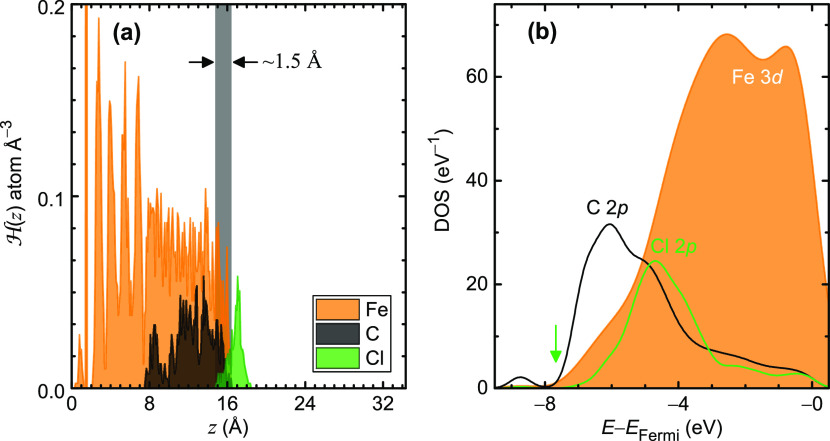
(a) Histogram  showing the density profile of the dissolution process at *t* = 16 ps. (b) Partial density of states (DOS) for the final equilibrated structure of polychloroprene dissolved in molten iron presented with respect to the Fermi level (*E*_Fermi_).

The final structure in Figure 3b and the radial distribution function in Figure 2b already demonstrate the exclusive C diffusion into the molten iron out of decomposed polychloroprene. However, these Figures are based on static snapshots from the end of the molecular dynamics run and do not provide any information about the local thermal fluctuations after the structure is equilibrated. A time-dependent insight can be obtained by examining the histogram of element-specific density profiles  sampled over consecutive bins. These bins slice the supercell into regions parallel to the surface of the molten iron, covering the entire supercell.  is calculated as
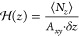
1Here, ⟨*N*_*z*_⟩ is the time-averaged number of atoms of a given species in a specific bin of the length δ*z* over the sampling time interval and *A*_*xy*_ is the supercell’s cross section. For reliable , δ*z* should be narrow enough so the density variation within any bin is imperceptible compared to the density variation across the supercell’s length. In our case, δ*z* was set as 0.1 Å. We also used 100 consecutive geometries obtained from a secondary molecular dynamics run at time steps of 0.05 fs performed on the equilibrated structure for averaging over time. This time step was an order of magnitude smaller than the one used for the dissolution simulation to avoid averaging over the dissolution process. The calculated histogram is shown in Figure 4a.

The histogram in Figure 4a, which shows the whereabouts of the atomic species over a time interval, indicates that the overwhelming majority of the C atoms remains below the molten iron surface after dissolution. C atoms diffuse ∼8 Å deep into the molten iron and do not show any significant presence above the surface. Cl’s histogram, however, exhibits a peak at *z* = ∼17 Å above the molten iron’s surface and has only the end tail of its histogram peak penetrating into the molten iron. Consequently, the time-averaged histogram once again validates the exclusive C dissolution into the molten iron even when thermal fluctuations are considered.

Electronically, the density of states (DOS) in liquids, because of the lack of translational symmetry, is generally more complex compared to that in crystalline materials. As a result, the DOS, in molten metals, is characterized by a continuous and broad distribution of states instead of well-defined energy bands and band gaps common in solid metals and alloys, making interpreting the results challenging. We, nonetheless, attempt to analyze the partial DOS of the equilibrated structure in Figure 3b. According to Figure 4b, the Fe 3*d* states constitute two gentle and broad peaks with maximum values above ∼ – 4 eV, tapering off in lower energies. This DOS pattern agrees well with earlier liquid iron simulations using different supercell sizes.^41^ Remarkably, this electronic behavior is in stark contrast to the Fe DOS in solid, which forms a very sharp peak closer to the Fermi level at ∼ – 0.5 eV.^42^ C 2*p* states peak at an even lower energy of ∼ – 6 eV in an outspread peak, contracting the DOS distribution in solid C where at the same energy, the DOS peak is considerably steeper and more pointed.^43^ Cl 2*p* states subside at a faster rate than C’s states (marked with green arrow), indicating a more electronic localization. The absence of abrupt variations or splitting in the partial DOS bands demonstrates a lack of specific short order or interaction among the atomic species after dissolution. The more localized Cl 2*p* states, compared to C 2*p* state, may also indicate spatial confinement,^44,45^ agreeing with the fact that the Cl atoms remain on the molten iron surface after the decomposition of polychloroprene.

The *g*(***r***), histogram, and DOS analyses indicated that polychloroprene could be an acceptable carburizing agent in steelmaking as its Cl content does not diffuse into molten iron but instead accumulates on the surface of molten iron, probably with the rest of the slag materials, eventually bonding to the byproduct cations. We can apply the Noyes–Whitney diffusion equation at the nanoscale to obtain a value for the carbon diffusivity (*D*) from polychloroprene into molten iron. The Noyes–Whitney equation reads:
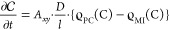
2where  is the rate of dissolution of polychloroprene’s carbon into molten iron, *A*_*xy*_ is the simulation supercell’s cross-section, *l* is the thickness of the interfacial layer, and ϱ_PC_(C) and ϱ_MI_(C) are the carbon density of the polychloroprene and molten iron, respectively.  can be approximated by .  is the number of completely dissolved C atoms determined to be those C atoms fully coordinated by Fe atoms after the structure equilibrate, i.e., 17 out of the 20 carbon atoms. Δ*t* is the time required for this dissolution to occur. These 17 C atoms were dissolved in molten iron by *t* = 10.003 ps. *l* can also be approximately equated to the depth at the interface where all chemical species, C, Cl, and Fe, moved through during the dissolution process, marked with two black arrows in Figure 4a. Solving eq 2 for *D* using these parameters yields *D* = 8.983 × 1 × 10^–5^ cm^2^ s^–1^. The experimentally measured value for C diffusivity from solid carbon is lower at 7.9 × 10^–5^ cm^2^ s^–1^.^46^

One should note that this calculated *D* value is obtained at the nanoscale and can not be directly compared to the values at the macroscale. At industrial scales, additional factors, such as temperature fluctuations, pH, and the presence of other substances, such as pigments in the polymer or alloying metals in molten iron, can reduce the observed diffusivity. However, the diffusivity predicted by our models can be compared with values obtained in similar models. For instance, similar *ab initio* molecular dynamics calculations predicted carbon diffusivity *D* = 6.00 × 10^–5^ cm^2^ s^–1^ for polycarbonate,^35^*D* = 1.368 × 10^–5^ cm^2^ s^–1^ for polysulfide, and *D* = 1.050 × 10^–5^ cm^2^ s^–1^ for polyurethane at *T* = 1550 °C,^36^ all substantially smaller than polychloroprene’s *D*. Benchmarks against these experimental and theoretical values, therefore, demonstrate the suitability of polychloroprene as a carburizing agent in steelmaking.

One last analysis we can obtain from our result is the volume expansion of molten iron after carbon dissolution. As seen in Figure 3b, the average position of the last Fe layer in the molten iron rose by 0.62 Å (denoted by Δ*z*) upon the complete diffusion of 17 C atoms out into the bulk region of the molten iron. This constitutes a 4.33% volume increase for alloying 2.70% carbon, measured by mass ratio at *T* = 1550 °C. Volume changes due to mass diffusion in liquid metals and alloys are, both experimentally and theoretically, challenging to characterize.^47^ Therefore, this minute piece of theoretical insight might be helpful in future developments.

Finally, we draw the reader’s attention to the potential future possibilities of similar simulation works in obtaining atomistic insight into large industrial processes. The simulation presented here consumed a considerable amount of ∼30 000 CPU hours on modern 64-core Intel CPUs. Despite the enormous resources needed for this simulation, the ***N***^3^ scalability of DFT formalism^48^ restricted our work in two ways, the system size and the time interval of the molecular dynamics run. Classical molecular dynamics simulations can overcome these restrictions as they scale as ***N***^1^ ∼ ***N***^2^ with the system size and are generally computationally more affordable.^49^ Yet obtaining reliable high-temperature force fields for any set of elements is not always possible,^50^ limiting the applicability of classical molecular dynamics to chemically diverse systems. However, recent machine-learning techniques allow the development of accurate classical force fields based on DFT calculations, acting as training sets.^51,52^ Developing these machine-learning-based classical force fields allows the extrapolation of our simulation to more complex systems, e.g., containing multiple types of carbonaceous materials and the resulting gases, or over longer time spans, e.g., in orders of nanoseconds or microseconds. In this regard, the recently developed DeePMD-kit package, which relies on TensorFlow, a robust and common deep learning framework, can be used to parametrize the potentials for accurate and lengthy molecular dynamics runs.^53,54^ Notably, the DFT calculations presented here can train next-generation classical force fields.

## Conclusions

Our *ab initio* molecular dynamics showed the preferential C dissolution into molten iron when in contact with polychloroprene. The polymer’s Cl atoms were predicted to remain at the molten iron’s surface after decomposition at *T* = 1550 °C. This insight hints at the suitability of polychloroprene as a carburizing agent in the steelmaking process, as chlorine, which is detrimental to steel’s mechanical properties, does not diffuse into molten iron and, consequently, would not form an alloy with the steel products. More rigorous analysis of the results using the Noyes-Whitney diffusion model also predicts that polychloroprene is a more efficient carburizing agent than other common polymers such as polycarbonate, polyurethane, and polysulfide commonly found in waste plastics. More importantly, our results indicate that no preprocessing or separation process is required to extract polychloroprene from mixed waste plastics before use as a carburizing agent. Furthermore, since our simulation explicitly only considered the diffusion competition between C and Cl in molten iron, the results can probably be generalized to other chlorinated hydrocarbon polymers, such as polyvinyl chloride, poly(*p*-vinylbenzyl chloride), or chlorinated polystyrene. Finally, the methodology presented here can be utilized in broader applications, such as investigating the preferential diffusivity of multiple atomic species in a given liquid in general, including other molten metals. Therefore, the methodology presented here can be applied to the processes of extracting or alloying other metals.

## Supporting Information Available

Trajectory of the secondary molecular dynamics run, used in Figure 4a, in VASP’s XDATCAR format, along with an explanatory note (ZIP)

## References

[ref1] Calvert P. Natural and synthetic polymers. Nature 1975, 253, 504–504. 10.1038/253504b0.

[ref2] Tarrahi R.; Fathi Z.; Seydibeyoğlu M. O.; Doustkhah E.; Khataee A. Polyhydroxyalkanoates (PHA): From production to nanoarchitecture. Int. J. Biol. Macromol. 2020, 146, 596–619. 10.1016/j.ijbiomac.2019.12.181. 31899240

[ref3] Maleki F.; Dasgupta P. K. Moldable strong cation exchange polymer and microchannel fabrication. Anal. Chem. 2020, 92, 13378–13386. 10.1021/acs.analchem.0c02754. 32862638

[ref4] Tiseo I. Annual production of plastics worldwide from 1950 to 2020. https://www.statista.com/statistics/282732/global-production-of-plastics-since-1950/, 2021.

[ref5] Charles D.; Kimman L. Plastic Waste Makers Index 2023; 2023. https://cdn.minderoo.org/content/uploads/2023/02/04205527/Plastic-Waste-Makers-Index-2023.pdf..

[ref6] The Organisation for Economic Co-operation and Development. Plastic Pollution is Growing Relentlessly as Waste Management and Recycling Fall Short, Says OECD. https://www.oecd.org/environment/plastic-pollution-is-growing-relentlessly-as-waste-management-and-recycling-fall-short.htm, 2022.

[ref7] Garforth A. A.; Ali S.; Hernández-Martínez J.; Akah A. Feedstock recycling of polymer wastes. Curr. Opin. Solid State Mater. Sci. 2004, 8, 419–425. 10.1016/j.cossms.2005.04.003.

[ref8] Seghar S.; Asaro L.; Aït Hocine N. Experimental validation of the Horikx theory to be used in the rubber devulcanization analysis. J. Polym. Environ. 2019, 27, 2318–2323. 10.1007/s10924-019-01513-z.

[ref9] Coates G. W.; Getzler Y. D. Chemical recycling to monomer for an ideal, circular polymer economy. Nat. Rev. Mater. 2020, 5, 501–516. 10.1038/s41578-020-0190-4.

[ref10] Sathe D.; Zhou J.; Chen H.; Su H.-W.; Xie W.; Hsu T.-G.; Schrage B. R.; Smith T.; Ziegler C. J.; Wang J. Olefin metathesis-based chemically recyclable polymers enabled by fused-ring monomers. Nat. Chem. 2021, 13, 743–750. 10.1038/s41557-021-00748-5. 34294914

[ref11] Puthiaraj P.; Lee Y.-R.; Ahn W.-S. Microporous amine-functionalized aromatic polymers and their carbonized products for CO_2_ adsorption. Chem. Eng. J. 2017, 319, 65–74. 10.1016/j.cej.2017.03.001.

[ref12] Doustkhah E.; Esmat M.; Fukata N.; Ide Y.; Hanaor D. A.; Assadi M. H. N. MOF-derived nanocrystalline ZnO with controlled orientation and photocatalytic activity. Chemosphere 2022, 303, 134932 10.1016/j.chemosphere.2022.134932. 35568217

[ref13] Sadeghi Rad T.; Ansarian Z.; Khataee A.; Vahid B.; Doustkhah E. N-doped graphitic carbon as a nanoporous MOF-derived nanoarchitecture for the efficient sonocatalytic degradation process. Sep. Purif. Technol. 2021, 256, 117811 10.1016/j.seppur.2020.117811.

[ref14] Hassandoost R.; Kotb A.; Movafagh Z.; Esmat M.; Guegan R.; Endo S.; Jevasuwan W.; Fukata N.; Sugahara Y.; Khataee A.; Yamauchi Y.; Ide Y.; Doustkhah E. Nanoarchitecturing bimetallic manganese cobaltite spinels for sonocatalytic degradation of oxytetracycline. Chem. Eng. J. 2022, 431, 133851 10.1016/j.cej.2021.133851.

[ref15] Choe Y. J.; Kim S. H.; Jeong K.; Kim J. Steering ^.OH-triggered radicalization of surface phosphate functionality and its protonated analogues to accelerate mineralization of aqueous organic wastes. Chem. Eng. J. 2023, 455, 140537 10.1016/j.cej.2022.140537.

[ref16] Bohlmann G. M. Biodegradable packaging life-cycle assessment. Environ. Prog. 2004, 23, 342–346. 10.1002/ep.10053.

[ref17] Can microbes save the planet? Nat. Biotechnol. 2023, 41, 735–735. 10.1038/s41587-023-01837-1 37264217

[ref18] Tan K. Q.; Ahmad M. A.; Oh W. D.; Low S. C. Valorization of hazardous plastic wastes into value-added resources by catalytic pyrolysis-gasification: A review of techno-economic analysis. Renew. Sustain. Energy Rev. 2023, 182, 113346 10.1016/j.rser.2023.113346.

[ref19] Panahi A.; Wei Z.; Song G.; Levendis Y. A. Influence of stainless-steel catalyst substrate type and pretreatment on growing carbon nanotubes from waste postconsumer plastics. Ind. Eng. Chem. Res. 2019, 58, 3009–3023. 10.1021/acs.iecr.8b05770.

[ref20] Neuschütz D. State and trends of the electric arc furnace technology. High Temp. Mater. Process. 2000, 4, 12 10.1615/HighTempMatProc.v4.i1.80.

[ref21] Echterhof T. Review on the Use of Alternative Carbon Sources in EAF Steelmaking. Metals 2021, 11, 222 10.3390/met11020222.

[ref22] Mansuri I.. Recycling waste polymers as a source of carbon in steelmaking: Fundamental high temperature investigations on structure evolution and carbon dissolution into molten iron. Ph.D. Thesis, University of New South Wales, Sydney, Australia, 2015.

[ref23] Königsberger E.; Spahiu K.; Herschend B. Thermodynamic Study of the Chlorine Content of Stainless Steel. Metall. Mater. Trans. B 2021, 52, 840–853. 10.1007/s11663-021-02057-1.

[ref24] Biggiero G.; Luzzi G. The study of mobile impurities in steels by Auger spectroscopy. Vacuum 1983, 33, 723–726. 10.1016/0042-207X(83)90598-5.

[ref25] Dwivedi D.; Lepková K.; Becker T. Carbon steel corrosion: a review of key surface properties and characterization methods. RSC Adv. 2017, 7, 4580–4610. 10.1039/C6RA25094G. PMC5378249 28413351

[ref26] Tsugita Y. Problems and prospects of halogen element contained dust treatment in recycling. Mater. Trans. 2003, 44, 2422–2426. 10.2320/matertrans.44.2422.

[ref27] Kaminsky W.; Mennerich C.; Andersson J.; Götting S. Pyrolysis of polychloroprene rubber in a fluidised-bed reactor - product composition with focus on chlorinated aromatic compounds. Polym. Degrad. Stab. 2000, 71, 39–51. 10.1016/S0141-3910(00)00147-6.

[ref28] Kresse G.; Hafner J. *Ab initio* molecular-dynamics simulation of the liquid-metal–amorphous-semiconductor transition in germanium. Phys. Rev. B 1994, 49, 14251–14269. 10.1103/PhysRevB.49.14251. 10010505

[ref29] Kresse G.; Joubert D. From ultrasoft pseudopotentials to the projector augmented-wave method. Phys. Rev. B 1999, 59, 1758–1775. 10.1103/PhysRevB.59.1758.

[ref30] Nosé S. A unified formulation of the constant temperature molecular dynamics methods. J. Chem. Phys. 1984, 81, 511–519. 10.1063/1.447334.

[ref31] Hoover W. G.; Holian B. L. Kinetic moments method for the canonical ensemble distribution. Phys. Lett. A 1996, 211, 253–257. 10.1016/0375-9601(95)00973-6.

[ref32] Blöchl P. E. Projector augmented-wave method. Phys. Rev. B 1994, 50, 17953–17979. 10.1103/PhysRevB.50.17953. 9976227

[ref33] Perdew J. P.; Chevary J. A.; Vosko S. H.; Jackson K. A.; Pederson M. R.; Singh D. J.; Fiolhais C. Atoms, molecules, solids, and surfaces: Applications of the generalized gradient approximation for exchange and correlation. Phys. Rev. B 1992, 46, 6671–6687. 10.1103/PhysRevB.46.6671. 10002368

[ref34] Haas P.; Tran F.; Blaha P.; Schwarz K.; Laskowski R. Insight into the performance of GGA functionals for solid-state calculations. Phys. Rev. B 2009, 80, 195109 10.1103/PhysRevB.80.195109.

[ref35] Assadi M. H. N.; Sahajwalla V. Recycling end-of-life polycarbonate in steelmaking: Ab initio study of carbon dissolution in molten iron. Ind. Eng. Chem. Res. 2014, 53, 3861–3864. 10.1021/ie4031105.

[ref36] Assadi M. H. N.; Sahajwalla V. Polymers’ surface interactions with molten iron: A theoretical study. Chem. Phys. 2014, 443, 107–111. 10.1016/j.chemphys.2014.09.007.

[ref37] Ramin L.; Assadi M. H. N.; Sahajwalla V. High-density polyethylene degradation into low molecular weight gases at 1823 K: An atomistic simulation. J. Anal. Appl. Pyrolysis 2014, 110, 318–321. 10.1016/j.jaap.2014.09.022.

[ref38] Zhou Y.; Luo G.; Hu Y.; Wu D.; Yao Z. Interaction properties between molten metal and quartz by molecular dynamics simulation. J. Mol. Liq. 2021, 342, 117474 10.1016/j.molliq.2021.117474.

[ref39] Yan H.-J.; Liu L.; Zhuang J.-C.; Zhou P.; Zhou C. Q. Molecular Dynamics Simulation of Carbon Effect on the Thermal Physical Properties of the Molten Iron. ISIJ. Int. 2019, 59, 221–226. 10.2355/isijinternational.ISIJINT-2018-513.

[ref40] Fruchart D.; Chaudouet P.; Fruchart R.; Rouault A.; Senateur J. Etudes structurales de composés de type cémentite: Effet de l’hydrogène sur Fe_3_C suivi par diffraction neutronique. Spectrométrie Mössbauer sur FeCo_2_B et Co_3_B dopés au^57^Fe. J. Solid State Chem. 1984, 51, 246–252. 10.1016/0022-4596(84)90340-2.

[ref41] González L. E.; González D. J. Structure and dynamics in liquid iron at high pressure and temperature. A first principles study. J. Geophys. Res. Solid Earth 2023, 128, e2022JB025119 10.1029/2022JB025119.

[ref42] Pou P.; Flores F.; Ortega J.; Pérez R.; Yeyati A. L. Electron correlation effects and ferromagnetism in iron. J. Condens. Matter Phys. 2002, 14, L421–L427. 10.1088/0953-8984/14/23/103.

[ref43] Charlier J.-C.; Gonze X.; Michenaud J.-P. First-principles study of the stacking effect on the electronic properties of graphite(s). Carbon 1994, 32, 289–299. 10.1016/0008-6223(94)90192-9.

[ref44] Roduner E. Size matters: why nanomaterials are different. Chem. Soc. Rev. 2006, 35, 583–592. 10.1039/b502142c. 16791330

[ref45] Fronzi M.; Bishop J.; Martin A. A.; Assadi M. H. N.; Regan B.; Stampfl C.; Aharonovich I.; Ford M. J.; Toth M. Role of knock-on in electron beam induced etching of diamond. Carbon 2020, 164, 51–58. 10.1016/j.carbon.2020.03.039.

[ref46] Morgan D.; Kitchener J. Solutions in liquid iron. Part 3:-Diffusion of cobalt and carbon. Trans. Faraday Soc. 1954, 50, 51–60. 10.1039/TF9545000051.

[ref47] Meyer A.; Kargl F. Diffusion of Mass in Liquid Metals and Alloys-Recent Experimental Developments and New Perspectives. Int. J. Microgravity Sci. Appl. 2013, 30, 30–35.

[ref48] Hafner J. *Ab-initio* simulations of materials using VASP: Density-functional theory and beyond. J. Comput. Chem. 2008, 29, 2044–2078. 10.1002/jcc.21057. 18623101

[ref49] Plimpton S. Computational limits of classical molecular dynamics simulations. Comput. Mater. Sci. 1995, 4, 361–364. 10.1016/0927-0256(95)00037-1.

[ref50] Becker C. A.; Tavazza F.; Trautt Z. T.; de Macedo R. A. B. Considerations for choosing and using force fields and interatomic potentials in materials science and engineering. Curr. Opin. Solid State Mater. Sci. 2013, 17, 277–283. 10.1016/j.cossms.2013.10.001.

[ref51] Huang Y.; Kang J.; Goddard W. A.; Wang L.-W. Density functional theory based neural network force fields from energy decompositions. Phys. Rev. B 2019, 99, 064103 10.1103/PhysRevB.99.064103.

[ref52] Unke O. T.; Chmiela S.; Sauceda H. E.; Gastegger M.; Poltavsky I.; Schütt K. T.; Tkatchenko A.; Müller K.-R. Machine learning force fields. Chem. Rev. 2021, 121, 10142–10186. 10.1021/acs.chemrev.0c01111. 33705118 PMC8391964

[ref53] Wang H.; Zhang L.; Han J.; Weinan E. DeePMD-kit: A deep learning package for many-body potential energy representation and molecular dynamics. Comput. Phys. Commun. 2018, 228, 178–184. 10.1016/j.cpc.2018.03.016.

[ref54] Fronzi M.; Amos R. D.; Kobayashi R. Evaluation of Machine Learning Interatomic Potentials for Gold Nanoparticle—Transferability towards Bulk. Nanomaterials 2023, 13, 1832 10.3390/nano13121832. 37368262 PMC10303715

